# Association between Genetic Variants in DNA and Histone Methylation and Telomere Length

**DOI:** 10.1371/journal.pone.0040504

**Published:** 2012-07-11

**Authors:** Sangmi Kim, Christine G. Parks, Zongli Xu, Gleta Carswell, Lisa A. DeRoo, Dale P. Sandler, Jack A. Taylor

**Affiliations:** 1 Department of Medicine - Section of Hematology/Oncology, Georgia Health Sciences University Cancer Center, Augusta, Georgia, United States of America; 2 Epidemiology Branch, National Institute of Environmental Health Sciences, Research Triangle Park, North Carolina, United States of America; 3 Laboratory of Molecular Carcinogenesis, National Institute of Environmental Health Sciences, Research Triangle Park, North Carolina, United States of America; Bellvitge Biomedical Research Institute (IDIBELL), Spain

## Abstract

Telomere length, a biomarker of aging and age-related diseases, exhibits wide variation between individuals. Common genetic variation may explain some of the individual differences in telomere length. To date, however, only a few genetic variants have been identified in the previous genome-wide association studies. As emerging data suggest epigenetic regulation of telomere length, we investigated 72 single nucleotide polymorphisms (SNPs) in 46 genes that involve DNA and histone methylation as well as telomerase and telomere-binding proteins and DNA damage response. Genotyping and quantification of telomere length were performed in blood samples from 989 non-Hispanic white participants of the Sister Study, a prospective cohort of women aged 35–74 years. The association of each SNP with logarithmically-transformed relative telomere length was estimated using multivariate linear regression. Six SNPs were associated with relative telomere length in blood cells with p-values<0.05 (uncorrected for multiple comparisons). The minor alleles of *BHMT* rs3733890 G>A (p = 0.041), *MTRR* rs2966952 C>T (p = 0.002) and *EHMT2* rs558702 G>A (p = 0.008) were associated with shorter telomeres, while minor alleles of *ATM* rs1801516 G>A (p = 0.031), *MTR* rs1805087 A>G (p = 0.038) and *PRMT8* rs12299470 G>A (p = 0.019) were associated with longer telomeres. Five of these SNPs are located in genes coding for proteins involved in DNA and histone methylation. Our results are consistent with recent findings that chromatin structure is epigenetically regulated and may influence the genomic integrity of telomeric region and telomere length maintenance. Larger studies with greater coverage of the genes implicated in DNA methylation and histone modifications are warranted to replicate these findings.

## Introduction

Like most eukaryotic organisms, human chromosomes are capped with a 6 base pair telomeric repeat (-TTAGGG-) that helps prevent incomplete DNA replication and genomic degradation [1]. Telomeres shorten with each cell division, and this progressive shortening has been postulated to be a causal factor, or at least an indicator, of organismal aging [2]. Short telomeres in blood cells have been inversely related to chronological age, and age-related disorders such as hypertension [3] and cardiovascular disease [4], [5].

Telomere length exhibits wide variation between individuals [6] and may vary across populations [7]. Genetic variation may account for an appreciable fraction of the differences in telomere length, as twin studies estimated heritability of telomere length between 36–86% [8], [9], [10], [11], [12], [13]. However, the genetic basis of telomere length remains elusive. Among candidate SNPs identified in genome-wide association studies, only those in *TERC* have been extensively replicated [14], [15], [16]. Loci on chromosome 12p11.2 [13], [17] and chromosome 14q23.2 [11] identified using quantitative trait linkage analysis have not been replicated in other studies [11], [14], [15], [18].

Significant associations with telomere length have been also found for SNPs in *MEN1*, *MRE1A*, *RECQL5*, and *TNKS* in a study evaluating 43 telomere-associated genes such as genes encoding telomerase, shelterin proteins and proteins involved in DNA repair [19]. Although not included as a candidate pathway in this study [19], emerging data suggest that epigenetic modifications might be another regulatory mechanism of telomere length. In mouse models, knockout of histone methyltransferases [20] or of DNA methyltransferases [21] both have been shown to result in abnormal telomere elongation: Telomeric DNA repeats lack CpG sites and are not directly methylated, but subtelomeric DNA is heavily methylated and correlates with telomere length and telomeric recombination in human cancer cell lines [22].

The purpose of the present study is to investigate genetic variants in DNA and histone methylation as well as other telomere biology-associated proteins in relation to telomere length in blood cells.

## Results

Relative telomere length, estimated from the ratio of telomeric DNA relative to a single copy gene DNA (t/s ratio), ranged from 0.43 to 2.71 with an average of 1.25 among 989 women in the present study. Table 1 shows the associations between relative telomere length and 38 SNPs in genes involved in telomere biology and DNA damage response. Only one out of these SNPs (*ATM* rs1801516 G>A) was found to be associated with relative telomere length after adjustment for age and breast cancer diagnosis (p = 0.031 for recessive model). In contrast, we found suggestive evidence for an association with relative telomere length for five of 33 SNPs in genes involved in DNA and histone methylation pathways (*BHMT* rs3733890 [p = 0.041], *MTRR* rs2966952 [p = 0.002], *MTR* rs1805087 [p = 0.038], *EHMT2* rs558702 [p = 0.002] and *PRMT8* rs12299470 [p = 0.019]; Table 2).

**Table 1:** Association of SNPs in telomere biology and DNA damage response pathway with relative telomere length in blood cells.

Nearby Gene	Rs no.	Genotype substitution	MAF	Additive P	Recessive P	Dominant P
**ABL1**	rs3808814	G/A	0.087	0.928	0.931	0.933
	rs3824400	C/T	0.130	0.392	0.885	0.38
**ATF7IP**	rs11055989	A/G	0.274	0.496	0.74	0.48
	rs7312042	A/G	0.501	0.595	0.118	0.489
**ATM**	rs1801516	G/A	0.122	0.056	**0.031**	0.158
	rs3218673	CC	0.000			
**ATR**	rs2229033	C/G	0.013	0.725		0.725
**BICD1**	rs2630578	G/C	0.181	0.29	0.539	0.315
**BRCA1**	rs4986850	C/T	0.073	0.951	0.421	0.919
	rs4986852	C/T	0.016	0.973	0.967	0.965
**BRCA2**	rs28897727	G/T	0.007	0.081		0.081
**CHEK2**	rs738722	C/T	0.279	0.269	0.895	0.175
**FANCD2**	rs9875081	G/A	0.148	0.195	0.639	0.197
**MNAT1**	rs4151223	T/G	0.280	0.217	0.258	0.336
**MRE11A**	rs1270146	T/C	0.448	0.834	0.927	0.694
**PARP1**	rs3219062	G/T	0.006	0.400		0.4
**PIF1**	rs17802279	A/T	0.438	0.068	0.143	0.131
**PINX1**	rs13270447	G/T	0.052	0.705	0.854	0.716
	rs17711777	T/C	0.066	0.224	0.834	0.224
	rs6992312	T/G	0.057	0.621	0.703	0.56
**POLD2 (AEBP1)**	rs13898	A/G	0.043	0.348	0.374	0.408
**POT1**	rs6973812	C/T	0.405	0.843	0.821	0.911
**PRDM2**	rs11580688	G/A	0.281	0.981	0.594	0.797
**RAD1**	rs2069473	T/C	0.071	0.825	0.111	0.627
**RAD17**	rs1045051	T/G	0.308	0.758	0.952	0.72
**RAD50**	rs2240032	C/T	0.213	0.578	0.973	0.493
**RAD51L1**	rs4902571	C/T	0.202	0.26	0.556	0.272
	rs6573834	T/C	0.215	0.457	0.396	0.184
	rs8009876	C/T	0.169	0.667	0.41	0.401
	rs999737	C/T	0.212	0.748	0.939	0.677
**RAD54B**	rs3019146	C/T	0.349	0.11	0.439	0.093
**RBBP8**	rs7243256	A/C	0.227	0.854	0.928	0.854
**TERT||CLPTM1L**	rs2735940	A/G	0.478	0.097	0.236	0.128
**TEX14/LOC645545**	rs12946522	T/G	0.173	0.886	0.551	0.927
**TNKS**	rs6986755	G/A	0.056	0.372	0.352	0.433
**TP53**	rs8079544	C/T	0.056	0.941	0.326	0.778
**WRN**	rs1346044	T/C	0.259	0.647	0.628	0.415
	rs1800391	G/A	0.091	0.388	0.611	0.281
	rs2553256	T/C	0.377	0.653	0.435	0.94
	rs3024239	C/T	0.492	0.321	0.536	0.323

Analyses are adjusted for age (continuous variable) and breast cancer status.

Relative telomere length (t/s ratio) was logarithmically transformed.

**Table 2:** Association of SNPs in genes in single carbon metabolism and histone methylation pathway with relative telomere length in blood cells.

Nearby Gene	Rs no.	Genotype substitution	MAF	Additive P	Recessive P	Dominant P
**BHMT**	rs3733890	G/A	0.305	0.141	0.823	**0.041**
**C1orf167/MTHFR**	rs1537514	G/C	0.104	0.59	0.814	0.604
	rs3737967	G/A	0.045	0.472	0.463	0.388
**CBS**	rs234706	G/A	0.364	0.427	0.088	0.938
**DNMT3A**	rs13036246	T/C	0.473	0.167	0.07	0.607
	rs17745484	C/T	0.329	0.886	0.595	0.891
	rs7575625	A/G	0.428	0.819	0.974	0.713
**DNMT3B**	rs742630	C/G	0.417	0.394	0.051	0.772
**EHMT2**	rs7887	G/T	0.343	0.213	0.207	0.385
**EHMT2 (ZBTB12||C2)**	rs558702	G/A	0.106	**0.007**	0.297	**0.008**
**MAT1A**	rs1985908	A/G	0.314	0.381	0.6	0.4
	rs7081756	T/G	0.349	0.213	0.387	0.258
**MBD4**	rs140693	C/T	0.004	0.433		0.433
**MTHFD1**	rs2236225	G/A	0.447	0.967	0.531	0.632
**MTHFR**	rs1801131	T/G	0.321	0.617	0.208	0.925
	rs1801133	G/A	0.358	0.337	0.916	0.16
**MTR**	rs12354209	A/G	0.395	0.769	0.468	0.897
	rs1805087	A/G	0.183	**0.035**	0.318	**0.038**
**MTRR**	rs10380	C/T	0.100	0.19	0.535	0.21
	rs1532268	C/T	0.385	0.177	0.403	0.192
	rs1801394	G/A	0.446	0.422	0.516	0.512
**MTRR (FASTKD3)**	rs2966952	C/T	0.159	**0.01**	**0.002**	0.059
**PON1**	rs662	A/G	0.294	0.433	0.758	0.412
	rs854560	A/T	0.366	0.541	0.483	0.183
**PON1||PON3**	rs757158	C/T	0.426	0.243	0.479	0.242
**PRMT1 (BCL2L12)**	rs3745469	G/A	0.101	0.448	0.153	0.192
**PRMT8**	rs12299470	G/A	0.103	0.287	**0.019**	0.607
	rs7962508	A/G	0.163	0.697	0.759	0.598
	rs7972248	T/C	0.469	0.406	0.212	0.873
**SLC19A1**	rs1051266	A/G	0.417	0.64	0.357	0.965
**TCN2**	rs1131603	T/C	0.046	0.313	0.918	0.279
	rs1801198	C/G	0.452	0.543	0.831	0.447
	rs9606756	A/G	0.112	0.419	0.809	0.41

Analyses are adjusted for age (continuous variable) and breast cancer status.

Relative telomere length (t/s ratio) was logarithmically transformed.

For the six SNPs that were significantly associated with relative telomere length at α = 0.05, we further estimated multivariable-adjusted relative telomere length by genotype under different genetic models and report the model with smallest p-value (Table 3). The minor alleles of *BHMT* rs3733890 G>A, *MTRR* rs2966952 C>T and *EHMT2* rs558702 G>A were associated with shorter telomeres, while minor alleles of ATM rs1801516 G>A, *MTR* 1805087 A>G and *PRMT8* rs12299470 G>A were associated with longer telomeres. Age and lifestyle factors like obesity and smoking are known to be important determinants of telomere length. In our data, however, there were no significant associations of obesity or smoking with relative telomere length [23]. Age was significantly associated with telomere length, but there was no evidence of effect modification of the association between telomere length and individual SNPs by age groups (age <55 years vs. ≥55 years).

**Table 3:** Association of selected SNPs with relative telomere length in blood cells.

Gene	rs no.	Model	Genotype	No.	Adjustedt/s ratio	95% CI		P-value
**ATM**	rs180156	Recessive	GG/GA	936	1.18	1.16	1.20	0.031
			AA	28	1.32	1.19	1.45	
**BHMT**	rs3733890	Dominant	GG	460	1.20	1.17	1.23	0.041
			GA/AA	494	1.16	1.13	1.19	
**EHMT2 (ZBTB12||C2)**	rs558702	Dominant	GG	769	1.19	1.17	1.22	0.008
			GA/AA	191	1.13	1.09	1.17	
**MTR**	rs1805087	Dominant	AA	646	1.17	1.14	1.19	0.038
			AG/GG	318	1.21	1.18	1.25	
**MTRR (FASTKD3)**	rs2966952	Recessive	CC/CT	937	1.19	1.17	1.21	0.002
			TT	25	1.01	0.91	1.12	
**PRMT8**	rs12299470	Recessive	GG/GA	952	1.18	1.16	1.20	0.019
			AA	12	1.41	1.21	1.64	

Analyses are all adjusted for age (continuous) and breast cancer status.

Adjusted t/s ratios were estimated by exponentiating the log-transformed estimates.

## Discussion

A few SNPs have been related to telomere length, but other common genetic variations related to telomere length remain to be discovered. In the present study, we carried out an analysis of common genetic variations in candidate genes in relation to telomere length in blood cells, and observed suggestive evidence for associations with telomere length for several polymorphisms in genes involved in DNA and histone methylation. We found that women inheriting the variant allele of *BHMT* rs3733890, *MTRR* rs2966952 and *EHMT2* rs558702 had shorter telomeres, whereas women inheriting the variant alleles of *MTR* rs1805087 and *PRMT8* rs12299470 had longer telomeres.

Epigenetic modifications are associated with telomere length [24]. Telomeres are flanked by large blocks of heterochromatin, which stabilize repetitive DNA sequences by inhibiting recombination between homologous repeats [25]. DNA methylation and histone H3 methylation at lysine 9 are associated with repressed chromatin [26] and deregulation of epigenetic modifications have long been known to affect the integrity of the telomeric region [25]. Conversely, in the absence of telomerase the progressive shortening of telomeres results in a variety of epigenetic changes including increased histone acetylation, decreased histone methylation, and subtelomeric DNA methylation [24]. Together, these suggest that epigenetic changes and the regulation and maintenance of telomere length are intertwined processes.

We observed that rs12299470, located in intron 1 of *PRMT8*, is associated with long telomeres. PRMT8 belongs to a family of protein arginine methyltransferases (PRMTs) [27], and recognizes a glycin- and arginine-rich (GAR) motif as a preferred methylation site [28]. It was recently found that the shelterin component TRF2 contains the GAR motif, and deletion of PRMT1 promotes the formation of dysfunctional telomeres via inhibiting the binding of TRF2 to telomeric DNA [29]. The role of PRMT8 in telomere stability and function remains to be fully elucidated. However, it is interesting to note that PRMT8 was identified because of a high degree of sequence homology with PRMT1 [27].

Euchromatic histone-lysine N-methyltransferase 2 (EHMT2) is a key histone methyltransferase [30] and is known to be particularly important for histone methylation of euchromatin [30]. The *EHMT2* rs558702 is located in the 5′ flanking region of the *EHMT2* gene (4,862 bp upstream from transcriptional start position), and predicted by TFsearch (http://www.cbrc.jp/research/db/TFSEARCH.html) to be in a putative binding site of v-Myb or c-Myb transcription factors. Therefore, it is possible that the variant allele limits the binding of Myb transcription factors to its consensus site and reduces the *EHMT2* gene expression.

Enzymes of folate single-carbon metabolism play an essential role for the synthesis of DNA precursors and remethylation of homocysteine for S-adenosylmethionine (SAM)-dependent DNA methylation [31]. Among those enzymes are betaine:homocysteine methyltransferase (BHMT), methyltetrahydrofolate:homocysteine methyltransferase (MTR) and 5-methyltetrahydrofolate-homocysteine methyltransferase reductase (MTRR). In the present study, the *BHMT* rs3733890 G>A was associated with shorter telomere length, and the *MTR* rs1805087 A>G was related to longer telomeres. The *BHMT* rs3733890 is a missense mutation resulting in the conversion of an arginine residue to a glutamine residue at codon 239 in exon 6, although the variant allele does not appear to change in enzyme activity or homocysteine levels [32], [33]. However, carriers of the variant alleles have been reported to have favorable health profiles such as low prevalence of coronary artery diseases [32] and reduced risk of several congenital anomalies such as orofacial cleft [34] and neural tube defect [35]. The *MTR* rs1805087 is a missense change resulting in an amino acid substitution from aspartic acid to glycine at codon 919 in exon 26. The variant allele of this SNP has been associated with moderate increase of homocysteine levels [36].

We also observed shorter telomeres associated with the *MTRR* rs2966952 C>T. This SNP was selected in the present analysis because it is located at the 5′ flanking region of the *MTRR* gene (1187 bp upstream from transcriptional start position) and is predicted by TFSEARCH (http://www.cbrc.jp/research/db/TFSEARCH.html) to destroy the binding site of transcriptional factor C/EBPβ [37]. However, the SNP also leads to a lysine to arginine amino acid change at codon 56 in exon 2 of the *FASTKD3* gene, which encodes fast kinase domain-containing protein 3, a mitochondrial protein essential for cellular respiration [38]. Although the functional implication of this gene in telomere length is not known, it is possible that the observed association between rs2966952 and telomere length is mediated through effects on *FASTKD3* rather than *MTRR*.

Two SNPs in the *MTHFR* gene (rs1801131 and rs1801133) were not associated with relative telomere length. This finding is in agreement with a previous report showing a weak association between *MTHFR* 677C>T polymorphism (rs1801133) and longer telomeres only among those having lower than median plasma folate concentration but no overall association between the variant allele and telomere length in men [39]. Possible effect modification by plasma folate status could not be evaluated in the present study.

The *ATM* (ataxia telangiectasia mutated) gene encodes a protein kinase, ATM, that regulates a large number of proteins including the checkpoint kinases CHK1 and CHK2 [40], [41]. Induction of the checkpoint kinases is crucial for cell cycle arrest in response to DNA damage, and defective checkpoint responses can cause genomic instability and neoplastic transformation [40]. The present study found longer telomere length associated with the *ATM* rs1801516 G>A. The polymorphism is a missense mutation resulting in an amino acid change from aspartic acid to asparagine (dbSNP) that was predicted by PolyPhen [42] to be possibly damaging. However, a previous study by Mirabello *et al.* examined this SNP and 8 additional SNPs in the *ATM* gene, and did not find significant association with telomere length [19].

Several limitations in the present study should be discussed. First, as a large number of statistical tests were performed, our findings are particularly subject to type I (false-positive) error. However, we chose to report the p-values without correction for multiple comparisons because the SNPs in our study were not selected randomly but from candidate genes based on functional prediction using SIFT [43] and PolyPhen [42]. Still, the results need to be interpreted with caution given that none of the associations would have reached the same level of significance after adjustment for multiple comparisons. Second, some important candidate genes were not evaluated in this study. For example, H3–K9 methylatransferase, SUV39H1 and SUV39H2 were shown to be associated with the heterochromatin protein HP1 [44], and their absence resulted in modification of telomeric chromatin structure, and subsequent alteration in telomere length [20]. However, none of SNPs for these two genes met our criteria for selection. Lastly, we should point out that this study took place within a cohort enriched for a family history of breast cancer. While these women might have different allele frequencies or telomere lengths than a sample of the general population, we have no *a priori* reason to believe that specific characteristics of this population would impact the observed relationship of SNPs and telomere length. However, such an effect remains a possibility until verified in other general populations.

In conclusion, the present study found associations with telomere length for candidate SNPs (*BHMT* rs3733890, *MTRR* rs2966952, *EHMT2* rs558702, *MTR* rs1805087 and *PRMT8* rs12299470) that are implicated in DNA and histone methylation. These results support existing findings of epigenetic regulation of telomere length. These novel associations with telomere length require further replication in larger studies with more substantial genomic coverage as well as functional characterization of the variant alleles.

## Materials and Methods

### Ethics Statement

All individuals were informed about purposes, requirements, and rights as study participants. Written informed consent was obtained from all participating subjects prior to data collection. This project was approved by the Institutional Review Board of the National Institute of Environmental Health Sciences, NIH and the Copernicus Group Institutional Review Board.

### Study Population and Telomere Length Measurement

Data are from the Sister Study, which is a nationwide cohort study of environmental and genetic risk factors for breast cancer among women aged 35 to 74 years who have a sister with breast cancer [45]. A case-cohort analysis within the Sister Study was performed to examine the relationship between telomere length in blood cells and breast cancer risk in 342 incident breast cancer cases and 736 subcohort members who were randomly selected from 29,026 participants enrolled by June 1, 2007. Methods for relative telomere length measurement and characteristics of the study population have been previously described [23]. Briefly, genomic DNA was extracted from prospectively collected frozen blood samples using an Autopure LS (Qiagen) in the NIEHS Molecular Genetics Core Facility, and 10 ng of the extracted DNA was robotically aliquoted and plated in duplicate onto each of 4 replicate 384-well plates. Telomere length was determined as the ratio of telomere repeat copy number to single copy gene copy number (T/S ratio) relative to that of an arbitrary reference sample, using the monochrome multiplex quantitative PCR protocol. This method has been shown to give a high correlation (R^2^ = 0.84) with telomere length determined by the traditional Southern blot analysis [46]. Plates were run on a BioRad CFX384 (Hercules, CA) with the cycling parameters previously described [23]. A 5-point standard curve ranging from 1.9 to 75 ng in a 2.5-fold dilution series run was generated in each assay plate to estimate the value for each sample T (telomere) and S (albumin single copy) using Biorad CFX Manager software. Standard curve efficiencies for both primer sets were above 90%, and regression coefficients were at least 0.99 in all PCR runs. Plates were verified for overall quality control parameters. Average coefficient of variation (%CV) was 11% and intraclass correlation coefficient (ICC) of a single T/S ratio was 0.85. Individual estimates were obtained from the average of up to eight replicate T/S ratio values.

### Genotyping

As a part of our study of telomeres and breast cancer risk, we selected a broad group of candidate genes related to telomere biology such as genes encoding telomerase and telomere-binding proteins, DNA repair and cell cycle checkpoint proteins, and epigenetic regulators of chromatin structure. Candidate SNPs were selected using the SNPinfo GenePipe tool, a web-based SNP selection tool that can integrate GWAS results with SNP functional predictions and linkage disequilibrium (LD) information [47]. Briefly, a list of candidate genes (N = 140), including some previously linked to regulation and maintenance of telomere length, was first filtered against the Cancer Genetic Markers of Susceptibility (CGEMS) breast cancer genome-wide association study (GWAS) [48] results to exclude genes that showed no evidence of association with breast cancer (i.e., had no SNPs with p<0.05). However, some candidate genes that were very poorly represented in the CGEMS GWAS panel were retained even if they had no SNPs with p<0.05.We define poor representation having <20% of known common SNPs (as reported for that gene in dbSNP and with MAF ≥0.05) in high LD (r^2^≥0.8) with at least one SNP in the GWAS panel. We then used SNPinfo to select SNPs from the remaining set of candidate genes. In addition to those showing associations with breast cancer risk, SNPinfo enhances selection of SNPs with predicted functional effects that are in high LD with the GWAS panel.

A total of 72 SNPs on 46 genes were included in the final analysis. Genotyping was conducted by the NIEHS Molecular Genetics Core Facility, using a custom-designed Illumina GoldenGate genotyping panel. A total of 20 HapMap trios (20*3 = 60 samples) were genotyped to evaluate parent-parent-child (P-P-C) error. A total of 20 Sister Study sample duplicates were included to monitor replication error. Illumina BeadStudio genotyping software (version 1.6.3) was used to call genotypes. Individual genotypes with an Illumina GenCall (GC) score below 0.25 were assigned as missing. The overall call rate was 0.998. Both averaged P-P-C genotype error and averaged replication error were 0. The concordance between our genotype data and data in HapMap for the 20 HapMap trios was an average of 0.998 for each SNP.

### Statistical Analysis

Out of 1,078 women who had relative telomere length measurements in baseline samples, the current analysis was restricted to 989 non-Hispanic white women comprised of 325 incident breast cancer cases and 664 subcohort members. Relative telomere length was not associated with breast cancer in our data [23], and minor allele frequencies of the SNPs were highly correlated between cases and subcohort members (Pearson r = 0.9981).

Relative telomere length measurements were skewed to the right and therefore were logarithmically transformed. The association of each SNP with relative telomere length was estimated using linear regression models that included age as a continuous variable and breast cancer status. The model fit was evaluated comparing additive, dominant and recessive linear regression models. Reported P-values are nominal two-tailed p-values and have not been corrected for multiple comparisons. All analyses were performed using the Stata 10.0 (College Station, TX).

## References

[1] Harley CB, Futcher AB, Greider CW (1990). Telomeres shorten during ageing of human fibroblasts.. Nature.

[2] von Zglinicki T (2002). Oxidative stress shortens telomeres.. Trends in Biochemical Sciences.

[3] Demissie S, Levy D, Benjamin EJ, Cupples LA, Gardner JP (2006). Insulin resistance, oxidative stress, hypertension, and leukocyte telomere length in men from the Framingham Heart Study.. Aging Cell.

[4] Brouilette SW, Moore JS, McMahon AD, Thompson JR, Ford I (2007). Telomere length, risk of coronary heart disease, and statin treatment in the West of Scotland Primary Prevention Study: a nested case-control study.. The Lancet.

[5] Fitzpatrick AL, Kronmal RA, Gardner JP, Psaty BM, Jenny NS (2007). Leukocyte telomere length and cardiovascular disease in the cardiovascular health study.. Am J Epidemiol.

[6] Takubo K, Izumiyama-Shimomura N, Honma N, Sawabe M, Arai T (2002). Telomere lengths are characteristic in each human individual.. ExpGerontol.

[7] Hunt SC, Chen W, Gardner JP, Kimura M, Srinivasan SR (2008). Leukocyte telomeres are longer in African Americans than in whites: the National Heart, Lung, and Blood Institute Family Heart Study and the Bogalusa Heart Study.. Aging Cell.

[8] Slagboom PE, Droog S, Boomsma DI (1994). Genetic determination of telomere size in humans: a twin study of three age groups.. Am J HumGenet.

[9] Njajou OT, Cawthon RM, Damcott CM, Wu SH, Ott S (2007). Telomere length is paternally inherited and is associated with parental lifespan.. ProcNatlAcadSciUSA.

[10] Jeanclos E, Schork NJ, Kyvik KO, Kimura M, Skurnick JH (2000). Telomere Length Inversely Correlates With Pulse Pressure and Is Highly Familial.. Hypertension.

[11] Andrew T, Aviv A, Falchi M, Surdulescu GL, Gardner JP (2006). Mapping genetic loci that determine leukocyte telomere length in a large sample of unselected female sibling pairs.. Am J HumGenet.

[12] Atzmon G, Cho M, Cawthon RM, Budagov T, Katz M (2009). Genetic variation in human telomerase is associated with telomere length in Ashkenazi centenarians.. Proceedings of the National Academy of Sciences.

[13] Vasa-Nicotera M, Brouilette S, Mangino M, Thompson JR, Braund P (2005). Mapping of a Major Locus that Determines Telomere Length in Humans.. The American Journal of Human Genetics.

[14] Codd V, Mangino M, van der Harst P, Braund PS, Kaiser M (2010). Common variants near TERC are associated with mean telomere length.. Nat Genet.

[15] Levy D, Neuhausen SL, Hunt SC, Kimura M, Hwang SJ (2010). Genome-wide association identifies OBFC1 as a locus involved in human leukocyte telomere biology.. Proceedings of the National Academy of Sciences.

[16] Prescott J, Kraft P, Chasman DI, Savage SA, Mirabello L (2011). Genome-wide association study of relative telomere length.. PLoSOne.

[17] Mangino M, Brouilette S, Braund P, Tirmizi N, Vasa-Nicotera M (2008). A regulatory SNP of the BICD1 gene contributes to telomere length variation in humans.. Human Molecular Genetics.

[18] Mangino M, Richards JB, Soranzo N, Zhai G, Aviv A (2009). A genome-wide association study identifies a novel locus on chromosome 18q12.2 influencing white cell telomere length.. Journal of Medical Genetics.

[19] Mirabello L, Yu K, Kraft P, De Vivo I, Hunter DJ (2010). The association of telomere length and genetic variation in telomere biology genes.. Hum Mutat.

[20] Garcia-Cao M, O’Sullivan R, Peters AH, Jenuwein T, Blasco MA (2004). Epigenetic regulation of telomere length in mammalian cells by the Suv39h1 and Suv39h2 histone methyltransferases.. NatGenet.

[21] Gonzalo S, Jaco I, Fraga MF, Chen T, Li E (2006). DNA methyltransferases control telomere length and telomere recombination in mammalian cells.. Nat Cell Biol.

[22] Vera E, Canela A, Fraga MF, Esteller M, Blasco MA (2008). Epigenetic regulation of telomeres in human cancer.. Oncogene.

[23] Kim S, Sandler DP, Carswell G, De Roo LA, Parks CG (2011). Telomere length in peripheral blood and breast cancer risk in a prospective case-cohort analysis: results from the Sister Study.. Cancer Causes and Control.

[24] Blasco MA (2007). The epigenetic regulation of mammalian telomeres.. Nat Rev Genet.

[25] Grewal SI, Moazed D (2003). Heterochromatin and epigenetic control of gene expression.. Science.

[26] Stancheva I (2005). Caught in conspiracy: cooperation between DNA methylation and histone H3K9 methylation in the establishment and maintenance of heterochromatin.. BiochemCell Biol.

[27] Lee J, Sayegh J, Daniel J, Clarke S, Bedford MT (2005). PRMT8, a New Membrane-bound Tissue-specific Member of the Protein Arginine Methyltransferase Family.. Journal of Biological Chemistry.

[28] Najbauer J, Johnson BA, Young AL, Aswad DW (1993). Peptides with sequences similar to glycine, arginine-rich motifs in proteins interacting with RNA are efficiently recognized by methyltransferase(s) modifying arginine in numerous proteins.. The Journal of biological chemistry.

[29] Mitchell TR, Glenfield K, Jeyanthan K, Zhu XD (2009). Arginine methylation regulates telomere length and stability.. Molecular and Cellular Biology.

[30] Shinkai Y, Tachibana M (2011). H3K9 methyltransferase G9a and the related molecule GLP.. Genes Dev.

[31] Selhub J, Miller JW (1992). The pathogenesis of homocysteinemia: interruption of the coordinate regulation by S-adenosylmethionine of the remethylation and transsulfuration of homocysteine.. AmJ ClinNutr.

[32] Weisberg IS, Park E, Ballman KV, Berger P, Nunn M (2003). Investigations of a common genetic variant in betaine-homocysteine methyltransferase (BHMT) in coronary artery disease.. Atherosclerosis.

[33] Li F, Feng Q, Lee C, Wang S, Pelleymounter LL (2008). Human betaine-homocysteine methyltransferase (BHMT) and BHMT2: common gene sequence variation and functional characterization.. MolGenetMetab.

[34] Mostowska A, Hozyasz KK, Biedziak B, Misiak J, Jagodzinski PP (2010). Polymorphisms located in the region containing BHMT and BHMT2 genes as maternal protective factors for orofacial clefts.. EurJ Oral Sci.

[35] Boyles AL, Billups AV, Deak KL, Siegel DG, Mehltretter L (2006). Neural tube defects and folate pathway genes: family-based association tests of gene-gene and gene-environment interactions.. EnvironHealth Perspect.

[36] Harmon DL, Shields DC, Woodside JV, McMaster D, Yarnell JW (1999). Methionine synthase D919G polymorphism is a significant but modest determinant of circulating homocysteine concentrations.. GenetEpidemiol.

[37] Jin G, Huang J, Hu Z, Dai J, Tang R (2010). Genetic variants in one-carbon metabolism-related genes contribute to NSCLC prognosis in a Chinese population.. Cancer.

[38] Simarro M, Gimenez-Cassina A, Kedersha N, Lazaro JB, Adelmant GO (2010). Fast kinase domain-containing protein 3 is a mitochondrial protein essential for cellular respiration.. BiochemBiophysResCommun.

[39] Paul L, Cattaneo M, D’Angelo A, Sampietro F, Fermo I (2009). Telomere Length in Peripheral Blood Mononuclear Cells Is Associated with Folate Status in Men.. The Journal of Nutrition.

[40] Motoyama N, Naka K (2004). DNA damage tumor suppressor genes and genomic instability.. Current Opinion in Genetics & Development.

[41] von Zglinicki T, Saretzki G, Ladhoff J, d’Adda di Fagagna F, Jackson SP (2005). Human cell senescence as a DNA damage response.. Mechanisms of Ageing and Development.

[42] Sunyaev S, Ramensky V, Koch I, Lathe W, Kondrashov AS (2001). Prediction of deleterious human alleles.. Human Molecular Genetics.

[43] Ng PC, Henikoff S (2003). SIFT: Predicting amino acid changes that affect protein function.. Nucleic Acids Res.

[44] Martin C, Zhang Y (2005). The diverse functions of histone lysine methylation.. NatRevMolCell Biol.

[45] Weinberg CR, Shore DL, Umbach DM, Sandler DP (2007). Using Risk-based Sampling to Enrich Cohorts for Endpoints, Genes, and Exposures.. American Journal of Epidemiology.

[46] Cawthon RM (2009). Telomere length measurement by a novel monochrome multiplex quantitative PCR method.. Nucleic Acids Research.

[47] Xu Z, Taylor JA (2009). SNPinfo: integrating GWAS and candidate gene information into functional SNP selection for genetic association studies.. Nucleic Acids Res.

[48] Hunter DJ, Kraft P, Jacobs KB, Cox DG, Yeager M (2007). A genome-wide association study identifies alleles in FGFR2 associated with risk of sporadic postmenopausal breast cancer.. Nat Genet.

